# Long-Term Outcomes of Laparoscopic Sleeve Gastrectomy—a Single-Center, Retrospective Study

**DOI:** 10.1007/s11695-017-2795-2

**Published:** 2017-07-13

**Authors:** Piotr K. Kowalewski, Robert Olszewski, Maciej S. Walędziak, Michał R. Janik, Andrzej Kwiatkowski, Natalia Gałązka-Świderek, Krzysztof Cichoń, Jakub Brągoszewski, Krzysztof Paśnik

**Affiliations:** 1 Department of General Surgery, Military Institute of Medicine, Szaserów 128, 04-141 Warsaw, Poland; 2 Department of Geriatrics, National Institute of Geriatrics Rheumatology and Rehabilitation, Warsaw, Poland; 3 Department of Ultrasound, Institute of Fundamental Technological Research, Polish Academy of Sciences, Warsaw, Poland; 4 Department of Internal Medicine, Regional Hospital, Łęczna, Poland

**Keywords:** Bariatric surgery, Sleeve, Long-term follow-up, Comorbidities, GERD

## Abstract

**Introduction:**

Sleeve gastrectomy (LSG) is one of the most popular bariatric procedures. We present our long-term results regarding weight loss, comorbidities, and gastric reflux disease.

**Material and Methods:**

We identified patients who underwent LSG in our institution between 2006 and 2009. We revised the data, and the patients with outdated contact details were tracked with the national health insurance database and social media (facebook). Each of the identified patients was asked to complete an online or telephone survey covering, among others, their weight and comorbidities. On that basis, we calculated the percent total weight loss (%TWL) and percent excess weight loss (%EWL), along with changes in body mass index (ΔBMI). Satisfactory weight loss was set at >50% EWL (for BMI = 25 kg/m^2^). We evaluated type 2 diabetes (T2DM) and arterial hypertension (AHT) based on the pharmacological therapy. GERD presence was evaluated by the typical symptoms and/or proton pump inhibitor (PPI) therapy.

**Results:**

One hundred twenty-seven patients underwent LSG between 2006 and 2009. One hundred twenty patients were qualified for this study. Follow-up data was available for 100 participants (47 female, 53 male). Median follow-up period reached 8.0 years (from 7.1 to 10.7). Median BMI upon qualification for LSG was 51.6 kg/m^2^. Sixteen percent of patients required revisional surgery over the years (RS group), mainly because of insufficient weight loss (14 Roux-Y gastric bypass—LRYGB; one mini gastric bypass, one gastric banding). For the LSG (LSG group *n* = 84), the mean %EWL was 51.1% (±22.3), median %TWL was 23.5% (IQR 17.7–33.3%), and median ΔBMI was 12.1 kg/m^2^ (IQR 8.2–17.2). Fifty percent (*n* = 42) of patients achieved the satisfactory %EWL of 50%. For RS group, the mean %EWL was 57.8% (±18.2%) and median %TWL reached 33% (IQR 27.7–37.9%). Sixty-two percent (*n* = 10) achieved the satisfactory weight loss. Fifty-nine percent of patients reported improvement in AHT therapy, 58% in T2DM. After LSG, 60% (*n* = 60) of patients reported recurring GERD symptoms and 44% were treated with proton pomp inhibitors (PPI). In 93% of these cases, GERD has developed de novo.

**Conclusions:**

Isolated LSG provides fairly good effects in a long-term follow-up with mean %EWL at 51.1%. Sixteen percent of patients require additional surgery due to insufficient weight loss. More than half of the subjects observe improvement in AHT and T2DM. Over half of the patients complain of GERD symptoms, which in most of the cases is a de novo complaint.

## Introduction

Laparoscopic sleeve gastrectomy (LSG) was introduced as preliminary bariatric procedure for superobese patients, whose BMI exceeded 50 kg/m^2^ [1]. Its purpose was to achieve substantial weight loss, therefore lowering the rate of possible complications during the second, more extensive procedure, such as Duodenal Switch or Laparoscopic Roux-en-Y Gastric Bypass [2]. Yet the results of LSG concerning excessive weight-loss, co-morbidities, and postoperative shift in the American Society of Anesthesiologists (ASA) physical classification score have changed bariatric surgeons’ approach to LSG as a sole bariatric procedure [3–5]. The worldwide popularity of LSG increased from 4.5% of all bariatric procedures in 2008 to 37% in 2013, and it became the most popular operation in the USA in 2015 [6–8]. First results regarding the long-term outcomes of LSG are being published, yet there is still a need for longer follow-up (exceeding 5 years) with smaller lost-to-follow up ratio [9, 10]. Since our institution is the leading center for bariatric surgery in the country, with over 300 operations performed each year [11], we would like to present our results regarding long-term outcomes of LSG.

## Aim

The aim of this study was to evaluate long-term clinical outcomes of laparoscopic sleeve gastrectomy regarding weight loss, comorbidities, physical activity, and complaints of gastroesophageal reflux disease (GERD).

## Materials and Methods

The study was approved by our Institution’s Review Board.

### Participants

Our institution database was revised for the records of patients who underwent LSG as a one-stage procedure between 2006 (when we started our sleeve gastrectomy program) and 2009. We gathered the data on their weight, body mass index (BMI), and co-morbidities. The patients who did not fulfill their check-ins and whose personal data was outdated were tracked using the national health insurance database, or found and contacted privately using social media (Facebook) and its support groups. The rest of the patients were contacted by phone. Every participant filled out a questionnaire regarding their current weight, medical history, physical activity habits—expressed by regular (minimum three times-a-week), over 30-min exercise routine, and sedentary behavior, assessed by hours spent siting down on a daily basis.

### Evaluation of the Outcomes

To measure the effectiveness of the procedure, we calculated the percentage total weight loss (%TWL), percentage excess weight loss (%EWL), and change in body mass index (ΔBMI).

To obtain the excess weight (EW), we subtracted ideal body weight (IBW) for BMI of 25 kg/m^2^ from the weight before surgery. Satisfactory weight loss after the surgery was defined by the %EWL greater than 50%.

GERD presence was evaluated by the typical symptoms and/or proton pump inhibitor (PPI) therapy, according to the latest guidelines [12]. The questions regarding typical symptoms (such as postprandial heartburn, regurgitation, chronic cough) were included in the survey.

We evaluated type 2 diabetes (T2DM) and arterial hypertension (AHT), the two major obesity related comorbidities, based on the pharmacological therapy—whether it was ceased after the surgery, the doses or number of drugs administered changed, or no changes were observed [13].

### Statistical Methods

We performed the statistical analysis using “Statistica” software (StatSoft). Normality of the data was tested with Shapiro-Wilk test. Continuous variables were compared with the Student’s *t* test for normally distributed or Mann-Whitney U test for non-normally distributed data. Categorical variables were compared using the chi^2^ test. Statistical significance was set at *p* < 0.05.

### Surgical Technique

Every procedure was performed by laparoscopy with five trocars. The 36F bougie was used to calibrate the sleeve. The linear gastrectomy started 5–6 cm proximal to the pylorus and continued up to the gastroesophageal junction. In some cases, a running suture was used to reinforce the staple line—this decision was made by the surgeon based on the intraoperative view (visible bleeding etc.) and own experience.

## Results

### Participants

One hundred twenty-seven consecutive patients underwent LSG between January 2006 and December 2009. Three patients died because of non-procedure-related causes. Out of the remaining 124, we were unable to contact 24, mostly because of outdated telephone numbers and addresses. One hundred patients completed our survey. Therefore, the follow-up rate reached 80%. Our median follow-up period reached 8.0 years (ranging from 7.1 to 10.7 years).

Forty-seven participants were female, 53 were male. The median age upon surgery was 39 years, ranging from 17 to 64. Median BMI upon qualification for LSG was 51.6 kg/m^2^ (Table 1).

**Table 1:** Demographic data before surgery

	Value	% (SD) (range)
Gender (female/male)	47/53	47%/53%
Median age	39.0	(17–64)
Median BMI (kg/m^2^)	51.6	(35.9–72.0)
Mean weight (kg)	153.1	(±26.3)

### Surgery and Complications

Median operating time was 105 min (ranging from 45 to 350 min) and median length of stay lasted 5 days (IQR 4–6). A typical, uncomplicated LOS was 4 days. Fifty-one percent of patients had the sleeve staple line reinforced with running suture, based on the operating surgeon’s experience. The most common early complication was rhabdomyolysis which affected 13% of patients and was successfully treated with intravenous fluids and forced diuresis. It was diagnosed by asymptomatic elevation of serum muscle enzymes levels (which were routinely evaluated at the beginning of our LSG program). We did not observe any case of renal failure due to rhabdomyolysis. Three patients suffered from hemorrhages which required surgical revision. One patient had sepsis provoked by a gastric leakage, treated with drainage and endoprothesis. The major complication rate was 4%. No perioperative deaths were noted.

### Additional Bariatric Surgery

Sixteen percent (*n* = 16) of our participants required additional, revisional surgery over the years (RS group), mainly because of insufficient weight loss (94%). One patient required a conversion to Roux-Y gastric bypass (LRYGB) because of severe GERD symptoms. Fourteen patients had LRYGB (87%), one underwent a mini-gastric bypass (outside of our institution), and one had a gastric band placed.

### Weight Loss

LSG was the sole bariatric procedure for 84 patients. The mean %EWL was 51.1% (±22.3), median %TWL was 23.5% (IQR 17.7–33.3%) and median ΔBMI was 12.1 kg/m^2^ (IQR 8.2–17.2). Fifty percent (*n* = 42) achieved the satisfactory %EWL of 50%.

Sixteen patients underwent another bariatric procedure and their mean %EWL was 57.8% (±18.2%) and median %TWL reached 33% (IQR 27.7–37.9%). In this case, even more patients (62% *n* = 10) achieved the satisfactory weight loss.

There were no statistically significant differences in %EWL between the two groups (*p* > 0.05), yet we observed significant difference in %TWL and ΔBMI (*p* = 0.031 and *p* = 0.017, accordingly) (Table 2).

**Table 2:** Weight loss effects

	LSG GROUP (*n* = 84)	REVISIONAL SURGERY RS GROUP (*n* = 16)	*p* value
%EWL	51.1% (±22.3)	57.8% (±18.2)	0.257^a^
%TWL	23.5% (IQR 17.7–33.3%)	33% (IQR 27.7–37.9%)	*0.031* ^b^
ΔBMI	12.1 kg/m^2^ (IQR 8.2–17.2)	17.9 kg/m^2^ (IQR 14.8–18.6)	*0.017* ^b^

^a^Student’s *t* test

^b^Mann-Whitney U test

### Comorbidities

Before surgery, 55 patients were treated for AHT (LSG group *n* = 49; RS group *n* = 6). After sleeve gastrectomy, 28% did not require pharmacological therapy for AHT, 31% had their doses reduced, 8% had their doses increased, and 33% did not see any change in therapy.

Twenty-six patients suffered from T2DM. After LSG, 37% did not receive any medication for diabetes, 21% had their doses reduced, 11% had their doses increased and/or had to take insulin, and 32% did not notice any change in their treatment. Results for the RS-group are presented in Table 3.

**Table 3:** Comorbidities treatment after surgery

		LSG group (*n* = 84)	RS group (*n* = 16)
AHT		*n* = 49	*n* = 6
	*discontinued*	***14 (28%)***	1 (17%)
	*fewer doses*	***15 (31%)***	2 (33%)
	*no change*	16 (33%)	3 (50%)
	*increased doses*	4 (8%)	0
T2DM		*n* = 19	*n* = 3
	*discontinued*	***7 (37%)***	1 (33.3%)
	*fewer doses*	***4 (21%)***	1 (33.3%)
	*no change*	6 (32%)	1 (33.3%)
	*increased doses*	2 (11%)	0

*AHT* arterial hypertension, *T2DM* type 2 diabetes

Improvement in therapy = treatment discontinued or with fever doses

### GERD

After LSG, 60% (*n* = 60) of patients reported recurring GERD symptoms and 44% were treated with PPI. Only four participants complained of reflux before the surgery, which means that 93% of the cases developed de novo GERD. There is no statistically significant correlation between GERD symptoms and weight loss effect.

### Lifestyle and Quality of Life

Most of the patients included in the study were not physically active, with only 31% exercising regularly (at least three times a week; at least for 30 min). Median daily sedentary time reached 8 h (IQR 5.5–10). Sixty-three percent of our participants were smoking before the surgery and after the follow-up 33% were active tobacco users.

We did not assess the quality of life of patients with any particular form, yet we did ask if they regret their decision to undergo LSG. Ninety-six percent gave a negative answer and were satisfied with the outcomes.

## Discussion

Our institution started the sleeve gastrectomy program in 2006. Since the long-term follow-up studies are just being published and the follow-up is rarely longer than 8 years, we wanted to share our experience regarding LSG. We have managed to achieve a follow-up rate of 80%, which is higher than the rate desired for a 5 year follow-up [14]. Our median follow-up period reached 8 years, ranging from 7 to 10 years.

While discussing the demography of the participants, we were surprised by the almost even gender ratio, which was typically dominated by female participants in other studies and our previous observations regarding bariatric surgery [9, 15–17].

Our main results concerning weight loss are similar to those in other publications. Juodeikis et al. in their systematic review report the mean %EWL of 54.8% at 8 years [10]. Our reoperation rate (16%) was a bit lower than in the longer studies, since Arman et al. reported their rate at 31.7% [9] (rev 3 note 16). We believe that some of our patients who are not satisfied with their weight loss will be qualified for another procedure. Out of the various additional procedures possible after LSG, out consultants chose LRYGB based on their particular experience, and its positive impact on GERD and comorbidities. We did not consider duodenal switch, since we believe that it requires a very strict follow up (possible deficiencies), which is not currently possible in our national health insurance plan.

We reported 13 cases (13%) of rhabdomyolysis, which may seem high compering to other studies [18]. However, in our cases, it was mostly asymptomatic elevation of creatine kinase (CK) serum levels.

Regarding other major complications, our study did not reveal any perioperative death. Our major complication rate was similar to the results reported by Shi et al. in their systematic review [19].

The methods used to assess the remission rate for comorbidities were similar to the ones reported by Arman et al. [9]. Our results regarding treatment of T2DM and AHT were lower than the ones presented by Juodeikis et al., who reported 77.8% improvement for T2DM and 68.0% for AHT [10].

Our results regarding GERD symptoms may be the most concerning. Forty-four percent of patients were treated with PPI for de novo GERD, while 60% of patients suffered from GERD symptoms. Other authors reported lower number of postoperative GERD cases (from 10 to 26%) [10, 20]. Based on our results and other recent studies, we believe that additional, prospective research is required regarding this issue [21].

### Limitations

This study has several limitations. The follow-up survey was performed by telephone or online questionnaire, therefore may be affected by a recall bias. We were not able to perform invasive tests regarding GERD and could only base our results on presented symptoms and declared pharmacotherapy. We did not assess objectively the severity and frequency of GERD symptoms. Again, the impact on comorbidities was analyzed only by changes in therapy, prescribed by other physicians.

## Conclusions

Based on our results, LSG may be considered a fairly effective bariatric procedure, with mean %EWL of 51.1% at an 8-year, long-term follow-up. Forty-two percent of patients achieved the satisfactory weight loss of over 50% EWL only after LSG (rev. 2 note 3) and 16% of the patients required conversion to other procedure due to insufficient weight loss. This fact may urge on a more thorough and meticulous follow up. Fifty-nine percent of patients reported improvement in AHT therapy, 58% in T2DM. We conclude that there is a high rate of GERD symptoms after LSG, which requires further studies and may alter our perspective on patient selection and the procedure itself.
